# Correction: Naïve Bayes is an interpretable and predictive machine learning algorithm in predicting osteoporotic hip fracture in-hospital mortality compared to other machine learning algorithms

**DOI:** 10.1371/journal.pdig.0001771

**Published:** 2026-10-06

**Authors:** Jo-Wai Douglas Wang

In the Abstract, there is an error in the eighth sentence of the paragraph. The correct sentence is: Top performing models were support vector machines, random forests, naïve Bayes [NB], extreme gradient boosting, and logistic regression (AUROCs ranging 0.682 – 0.699, p > 0.05).

In the 2.2. Model performance–training subsection of the Results, there are errors in the first and second paragraph. The correct paragraphs are: The model with the highest area under the receiver operating characteristic (AUROC) was MLP (AUROC 0.828), followed by LR, RF, XGB, NB and SVM (0.733, 0.730, 0.726, 0.725 and 0.722 respectively, all p > 0.05), then DT (AUROC 0.697).

The model with greatest area under the precision-recall curve (AUPRC) was MLP (AUPRC 0.245), followed by LR, XGB and RF (AUPRCs of 0.134, 0.133 and 0.130 respectively, p > 0.05), NB (AUPRC 0.124), SVM (AUPRC 0.120) and DT (AUPRC 0.094). Details are present in Table 2 and Table 3.

In the 2.3. Model performance–test subsection of the Results, there are errors in the first and second paragraph. The correct paragraphs are: The models with highest AUROC were SVM, RF, NB, XGB and LR (AUROCs of 0.699, 0.696, 0.694, 0.689 and 0.682 respectively, all p > 0.05), followed by MLP and DT (AUROCs of 0.618 and 0.616 respectively, p > 0.05).

The models with highest AUPRC were SVM, NB, XGB, RF and LR (AUPRCs of 0.113, 0.113, 0.113, 0.112 and 0.112 respectively, p > 0.05), followed by MLP and DT (AUPRCs of 0.077 and 0.070 respectively, p > 0.05) – see Table 4 and Table 5.

In the 3.1. Model performance–training and test subsection of the Discussion, there is an error in the first paragraph. The correct paragraph is: Notably, but unsurprisingly, classification performance showed some variation among algorithms. The trained models, ordered in decreasing performance (based on both test AUROCs and test AUPRCs), were SVM, RF, NB, XGB and LR (all with no statistically significant difference in performance – see Tables 4 and 5, followed by MLP and DT (no statistically significant difference in performance). AUROCs ranged from 0.616 (DT) to 0.699 (SVM), while AUPRC values ranged from 0.070 (DT) to 0.113 (SVM, NB and XGB); a reflection of using a simplified model (with binary input data) to perform predictions on a minority class in this imbalanced dataset. There was minimal difference between the training and cross-validation performance for the top five models (SVM, RF, NB, XGB and LR). A greater variation in training and cross-validation performance scores was noted for DT and MLP, an indicator of overtraining (an infamous tendency in machine learning). That overtraining has occurred despite systematic and meticulous hyperparameter tuning, is strongly suggestive of insufficient data.

In the 2.4. Feature importance–Model interpretation subsection of the Results, there is an error in the second sentence of the second paragraph. The correct sentence is: In the corrected SVM model, the 5 most important patient features in prediction of mortality were CKD, hyperparathyroidism (PTH > 6.8pmol/L), CAD, advanced age (>80 years), and residence in a PRCF.

In Tables 2–5 errors were identified in the reported Support Vector Machine (SVM) metrics. Please see the correct Tables 2–5 here.

There are errors identified in the reported Support Vector Machine (SVM) metrics in S1 Fig and S3 Appendix, Table C. Please view the correct S1 Fig and S3 Appendix below.

**Table 2:** Model performance (training phase).

	AUROC			AUPRC		
	Mean	STD	95%CI	Mean	STD	95%CI
SVM	0.722	0.008	0.712–0.731	0.120	0.004	0.116–0.125
NB	0.725	0.007	0.711–0.739	0.124	0.003	0.117–0.131
LR	0.733	0.008	0.717–0.750	0.134	0.004	0.127–0.141
DT	0.697	0.004	0.690–0.704	0.094	0.001	0.093–0.095
RF	0.730	0.007	0.716–0.745	0.130	0.003	0.125–0.136
XGB	0.726	0.007	0.711–0.741	0.133	0.005	0.122–0.144
MLP	0.828	0.008	0.813–0.844	0.245	0.030	0.186–0.305

**Table 3:** Comparison of model performance during training: (A) AUROC; (B) AUPRC.

(A) AUROC														
	*t*-test statistic							*t*-test *p*-value						
Models	SVM	NB	LR	DT	RF	XGB	MLP	SVM	NB	LR	DT	RF	XGB	MLP
SVM	–	−0.722	−2.273	6.240	−1.784	−0.934	−21.218	–	0.491	0.053	0.000	0.112	0.378	0.000
NB	–	–	−1.683	7.766	−1.129	−0.226	−21.666	–	–	0.131	0.000	0.291	0.827	0.000
LR	–	–	–	9.000	0.631	1.472	−18.776	–	–	–	0.000	0.546	0.179	0.000
DT	–	–	–	–	−9.153	−8.043	−32.750	–	–	–	–	0.000	0.000	0.000
RF	–	–	–	–	–	0.904	−20.614	–	–	–	–	–	0.393	0.000
XGB	–	–	–	–	–	–	−21.456	–	–	–	–	–	–	0.000
MLP	–	–	–	–	–	–	–	–	–	–	–	–	–	–
**(B) AUPRC**														
	***t*-test statistic**							***t*-test *p*-value**						
Models	SVM	NB	LR	DT	RF	XGB	MLP	SVM	NB	LR	DT	RF	XGB	MLP
SVM	–	−1.686	−5.600	15.614	−4.525	−4.538	−9.216	–	0.130	0.001	0.000	0.002	0.002	0.000
NB	–	–	−4.472	21.213	−4.472	−3.451	−8.974	–	–	0.002	0.000	0.002	0.009	0.000
LR	–	–	–	21.693	2.236	0.349	−8.201	–	–	–	0.000	0.056	0.736	0.000
DT	–	–	–	–	−80.498	−17.103	−11.249	–	–	–	–	0.000	0.000	0.000
RF	–	–	–	–	–	−1.342	−8.572	–	–	–	–	–	0.217	0.000
XGB	–	–	–	–	–	–	−8.234	–	–	–	–	–	–	0.000
MLP	–	–	–	–	–	–	–	–	–	–	–	–	–	–

**Table 4:** Model performance during testing (five-fold cross-validation).

	AUROC			AUPRC - Average precision		
	Mean	STD	95%CI	Mean	STD	95%CI
SVM	0.699	0.027	0.666–0.733	0.113	0.019	0.089–0.137
NB	0.694	0.024	0.646–0.742	0.113	0.019	0.076–0.151
LR	0.682	0.034	0.614–0.750	0.112	0.019	0.074–0.150
DT	0.616	0.028	0.560–0.671	0.070	0.006	0.058–0.081
RF	0.696	0.030	0.636–0.756	0.112	0.024	0.064–0.161
XGB	0.689	0.025	0.640–0.738	0.113	0.024	0.065–0.160
MLP	0.618	0.027	0.565–0.671	0.077	0.007	0.063–0.090

**Table 5:** Comparison of model performance during testing: (A) AUROC; (B) AUPRC.

(A) AUROC														
	*t*-test statistic							*t*-test *p*-value						
Models	SVM	NB	LR	DT	RF	XGB	MLP	SVM	NB	LR	DT	RF	XGB	MLP
SVM	–	0.340	0.902	4.802	0.169	0.608	4.784	–	0.742	0.393	0.001	0.870	0.560	0.001
NB	–	–	0.645	4.729	−0.116	0.323	4.704	–	–	0.537	0.001	0.910	0.755	0.002
LR	–	–	–	3.351	−0.690	−0.371	3.296	–	–	–	0.010	0.509	0.720	0.011
DT	–	–	–	–	−4.359	−4.349	−0.115	–	–	–	–	0.002	0.002	0.911
RF	–	–	–	–	–	0.401	4.321	–	–	–	–	–	0.699	0.003
XGB	–	–	–	–	–	–	4.315	–	–	–	–	–	–	0.003
MLP	–	–	–	–	–	–	–	–	–	–	–	–	–	–
**(B) AUPRC**														
	***t*-test statistic**							***t*-test *p*-value**						
Models	SVM	NB	LR	DT	RF	XGB	MLP	SVM	NB	LR	DT	RF	XGB	MLP
SVM	–	0.013	0.103	4.843	0.085	0.055	4.025	–	0.990	0.920	0.001	0.934	0.958	0.004
NB	–	–	0.083	4.826	0.073	0.000	3.976	–	–	0.936	0.001	0.944	1.000	0.004
LR	–	–	–	4.713	0.000	−0.073	3.865	–	–	–	0.002	1.000	0.944	0.005
DT	–	–	–	–	−3.796	−3.887	−1.698	–	–	–	–	0.005	0.005	0.128
RF	–	–	–	–	–	−0.066	3.130	–	–	–	–	–	0.949	0.014
XGB	–	–	–	–	–	–	3.220	–	–	–	–	–	–	0.012
MLP	–	–	–	–	–	–	–	–	–	–	–	–	–	–

## Supporting information

S1 Fig ML model test performance (area under the receiver operating characteristic, AUROC). Only the test set AUCs evaluated from the 5-fold cross-validation for the five best-performing ML models are shown.
(PPTX)

S3 Appendix Supplementary data. (DOCX)
