# Supplementary material for: Naïve Bayes is an interpretable and predictive machine learning algorithm in predicting osteoporotic hip fracture in-hospital mortality compared to other machine learning algorithms
Source: PLOS Digit Health. 2025 Jan 2;4(1):e0000529. doi: 10.1371/journal.pdig.0000529 (PMC11694905; doi:10.1371/journal.pdig.0000529)
Supplement: S1 Fig — Only the test set AUCs evaluated from the 5-fold cross-validation for the four best-performing ML models are shown. (PPTX) [file pdig.0000529.s001.pptx]

## Slide 1
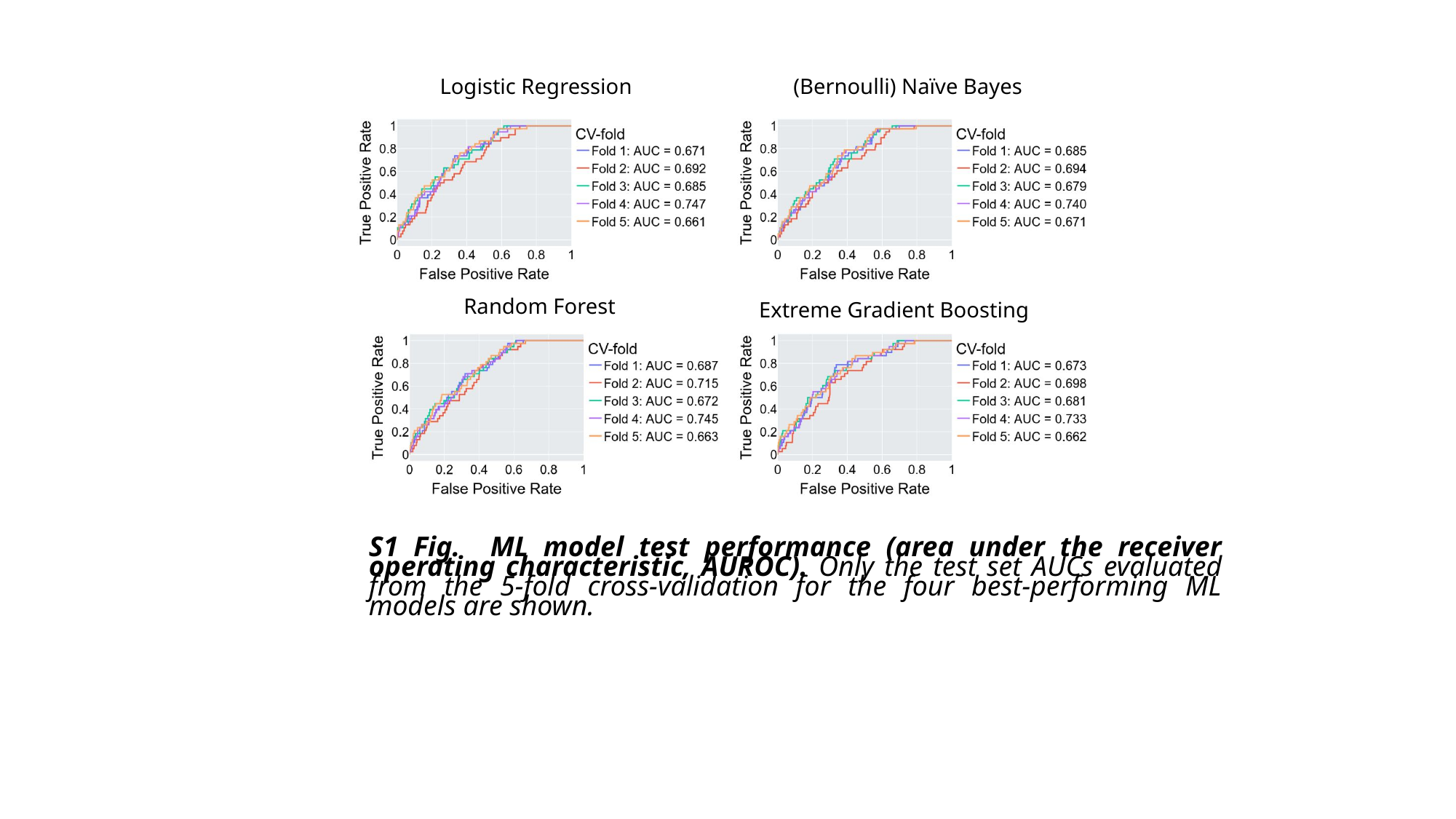

Logistic Regression
 (Bernoulli) Naïve Bayes
Random Forest
Extreme Gradient Boosting
S1 Fig. ML model test performance (area under the receiver operating characteristic, AUROC). Only the test set AUCs evaluated from the 5-fold cross-validation for the four best-performing ML models are shown.
