# Supplementary material for: Naïve Bayes is an interpretable and predictive machine learning algorithm in predicting osteoporotic hip fracture in-hospital mortality compared to other machine learning algorithms
Source: PLOS Digit Health. 2025 Jan 2;4(1):e0000529. doi: 10.1371/journal.pdig.0000529 (PMC11694905; doi:10.1371/journal.pdig.0000529)
Supplement: S1 Appendix — (DOCX) [file pdig.0000529.s002.docx]

# S1 Appendix

In the following section some mathematical context underlying the models is given and the approach used to obtain feature importance, for each model (RF, XGB, NB and LR) is shown. The average of the scores obtained during 5-fold cross-validation was used as the final feature score. The feature scores for NB and LR have the added benefit of offering intuitive insight as noted above in the discussion.

## LR (logistic regression).

The LR predicts a probability using the function:

$$f(x) = \frac{1}{1+exp(-\boldsymbol{x\cdot\beta-}\beta\boldsymbol{)}}$$

where $\boldsymbol{x}=[x_{1},x_{2},\ldots,x_{n}]$ is a vector for a patient encoding the presence or absence of a comorbidity (values of ‘1’ and ‘0’ respectively), $\boldsymbol{\beta=[}\beta_{\boldsymbol{1}},\beta_{\boldsymbol{2}},\beta_{\boldsymbol{3}},...,\beta_{\boldsymbol{n}}]$ is a vector containing the coefficient weights (corresponding, in this study, to each patient comorbidity) and $\beta$ the intercept value.

## (*Bernoulli*) Naïve Bayes (NB).

Naïve Bayes uses the following classification rule:

$$\hat{y}=\underset{y}{\mathrm{argmax}} P(y)\prod_{i=1}^{n} P(x_{i}|y)$$

In Bernoulli Naïve Bayes (i.e. for binary classification, using binarized input):

$$P\left( x_{i} | y \right)=P\left( x_{i}=1 | y \right)x_{i}+\left( 1-P\left( x_{i}=1 | y \right) \right)\left( 1-x_{i} \right)$$

For each patient comorbidity $x_{i}$, the model coefficient (i.e. the feature importance score) was computed as:

$$coefficients = \frac{P(x_{i}|y=1)}{P(x_{i}|y=0)}$$

where $P(x_{i}|y=1)$ and $P(x_{i}|y=0)$ is the probability of the $i^{th}$ comorbidity occurring given they experienced in-hospital mortality ($y=1$) and survived to discharge ($y=0$) respectively.

## Tree-based methods (DT, RF, XGB).

In developing a single decision tree, the features are recursively partitioned to group patients by outcome (mortality). At each step (or ‘node’) the feature that results in the greatest reduction in ‘impurity’, or conversely the greatest increase in ‘purity’ is chosen. The measured used in this paper for all tree-based methods (DT, XGB and RF) was the Gini impurity which is given by the formula:

$$H(Q_{m})=\sum_{k} p_{mk}(1-p_{mk})$$

Where $p_{mk}$ is the proportion of those who died (k=1) or survived (k=0) at decision step (or node) number $m$ and subsequently simplifies to:

$$H(Q_{m})=p_{m1}(1-p_{m1})+p_{m0}(1-p_{m0})$$

For RFs and XGBs where features may be used more than once (multiple trees are trained) the mean decrease in impurity across all nodes and trees is computed.
