# Supplementary material for: Naïve Bayes is an interpretable and predictive machine learning algorithm in predicting osteoporotic hip fracture in-hospital mortality compared to other machine learning algorithms
Source: PLOS Digit Health. 2025 Jan 2;4(1):e0000529. doi: 10.1371/journal.pdig.0000529 (PMC11694905; doi:10.1371/journal.pdig.0000529)
Supplement: S2 Appendix — (DOCX) [file pdig.0000529.s003.docx]

# S2 Appendix – SHAP analysis

In this section the principles underlying SHAP analysis are detailed.

SHAP values, based on concepts originating from cooperative game theory, are a unified measure of feature importance.

Formally speaking, they are the solutions to the following equation:

$$\varphi_{i}=\sum_{S\subseteq F\backslash\{i\}} \frac{\left| S \right|!\left( \left| F \right|-\left| S \right|-1 \right)!}{\left| F \right|!}[f_{S\cup\left\{ i \right\}}\left( x_{S\cup\left\{ i \right\}} \right)-f_{S}(x_{S})] (1)$$

where $\varphi_{i}$ is the SHAP value for the $i^{th}$ feature, $S$ represent subsets of all features and $F$ is the set of all features. The model trained with feature i present is denoted $f_{S\cup\left\{ i \right\}}$, the model trained with feature $i$ withheld is denoted $f_{S}$, with corresponding values $x_{S\cup\left\{ i \right\}}$ and $x_{S}$, representing the inputs in the set $S\cup\{i\}$ and $S$respectively. One can see that the SHAP value $\varphi_{i}$ is the weighted average of all possible differences between the models trained with and without feature i.

We used the Python implementation of SHAP (shap.Explainer, shap.KernelExplainer, and shap.TreeExplainer).

It is worth noting the exponential time complexity associated with computing SHAP values via equation (1). Indeed, for each feature i, two models are trained for all combination of features in the set of features without i, (i.e.$F\{i\}$). Direct computation with (1) is unfeasible and the Python implementations estimate SHAP values using sampling approximations (quasi-random sampling). Detailed discussions can be found in the literature, as referenced in the methods section [33, 34].

It should be noted that SHAP values cannot be used to make causal inferences.
