# Supplementary material for: Naïve Bayes is an interpretable and predictive machine learning algorithm in predicting osteoporotic hip fracture in-hospital mortality compared to other machine learning algorithms
Source: PLOS Digit Health. 2025 Jan 2;4(1):e0000529. doi: 10.1371/journal.pdig.0000529 (PMC11694905; doi:10.1371/journal.pdig.0000529)
Supplement: S3 Appendix — (DOCX) [file pdig.0000529.s004.docx]

# S3 Appendix – Supplementary Data

Table A. Hyperparameter search ranges.

| ML algorithm | Hyperparameters: search range |
| --- | --- |
| Logistic Regression | Penalty norm: none, L1, L2, L1 and L2  C (regularisation parameter): 0.1 – 100 |
| Support vector machines | Kernel: linear, radial basis function (rbf), sigmoid, polynomial  C (regularisation parameter): 0.1 - 100  polynomial degree (ignored if kernel ≠ ‘polynomial’): 3 – 10  gamma: 0.001 - 1 |
| Naive Bayes | alpha: 1.0  force_alpha: False  fit_prior: False |
| Decision Trees | Criterion: gini, entropy  max_depth: 1 - 100  min_samples_split: 10 - 100  min_samples_leaf: 10 - 100  max_features: 4 - 24 |
| Random Forest | criterion: gini, entropy  n_estimators: 100 - 1000  max_depth: 4 - 20  min_samples_split: 2 - 500  min_samples_leaf: 2 - 500  max_features: 4 – 24 |
| Extreme Gradient Boosting | n_estimators: 100 - 1000  learning_rate: 0.001 – 0.1  max_depth: 2 – 20  min_child_weight: 1 - 10  subsample: 0.01 – 1.0  colsample_bytree: 0.1 – 1.0  objective: binary:logistic |
| Multi-layer perceptron | number of hidden layers: 1 – 5  hidden layer sizes: 5 – 50  activation_layer: relu, tanh, logistic  alpha (regularisation parameter): 0.000001 – 0.01  solver: LBFGS (limited memory, Broyden-Fletcher-Goldfarb-Shanno algorithm)  Loss function: log-loss |

Table B. Proportion of patients with features undefined.

| Input Feature | Number of patients with data missing (n) | Percentage of patients with data missing (%) |
| --- | --- | --- |
| sex/gender | 0 | 0.00% |
| age | 0 | 0.00% |
| resident of PRCF | 0 | 0.00% |
| Smoker | **1** | **0.03%** |
| Alcohol overuse | **1** | **0.03%** |
| Walking aid use | **1** | **0.03%** |
| Hypertension | 0 | 0.00% |
| Coronary artery disease | **1** | **0.03%** |
| History of acute myocardial infarction | **1** | **0.03%** |
| Atrial fibrillation | 0 | 0.00% |
| Cerebrovascular accident | 0 | 0.00% |
| Transient ischaemic attack | **1** | **0.03%** |
| Dementia | **1** | **0.03%** |
| Parkinson’s Disease | **1** | **0.03%** |
| Chronic obstructive pulmonary disease | **1** | **0.03%** |
| Type 2 diabetes mellitus | 0 | 0.00% |
| Chronic Kidney Disease | **2** | **0.06%** |
| Anaemia | 0 | 0.00% |
| Cancer (active) | 0 | 0.00% |
| Osteoporosis | 0 | 0.00% |
| Parathyroid hormone > 6.8 | 0 | 0.00% |
| 25(OH)vitamin D ≤ 25 nmol/L | 0 | 0.00% |
| 25(OH)vitamin D ≤ 50 nmol/L | 0 | 0.00% |

**Table C.. Feature importance rankings.**

| Feature | Feature importance rankings* | | | | | | | |
| --- | --- | --- | --- | --- | --- | --- | --- | --- |
|  | LR | SVM | NB | DT | RF | XGB | MLP |  |
| Male | 9 | 6 | 13 | 12 | 12 | 12 | 3 |  |
| Aged > 80 | 3 | 1 | 12 | 5 | 5 | 3 | 14 |  |
| Resident of PRCF | 10 | 20 | 7 | 7 | 7 | 6 | 13 |  |
| Smoking | 13 | 17 | 20 | 15 | 15 | 19 | 23 |  |
| Alcohol overuse | 22 | 22 | 23 | 16 | 16 | 20 | 21 |  |
| Walking aids use | 16 | 5 | 14 | 17 | 17 | 13 | 6 |  |
| HT | 18 | 7 | 19 | 18 | 18 | 22 | 20 |  |
| CAD | 8 | 14 | 4 | 3 | 3 | 2 | 19 |  |
| MI | 7 | 8 | 1 | 8 | 8 | 7 | 5 |  |
| AF | 5 | 15 | 2 | 13 | 13 | 5 | 1 |  |
| CVA | 20 | 11 | 16 | 19 | 19 | 16 | 22 |  |
| TIA | 15 | 13 | 11 | 20 | 20 | 15 | 10 |  |
| dementia | 12 | 10 | 8 | 4 | 4 | 9 | 4 |  |
| PD | 14 | 19 | 18 | 21 | 21 | 17 | 18 |  |
| COPD | 4 | 12 | 6 | 11 | 11 | 11 | 12 |  |
| T2DM | 17 | 16 | 15 | 10 | 10 | 14 | 11 |  |
| CKD | 1 | 2 | 3 | 1 | 1 | 1 | 2 |  |
| Anaemia | 11 | 4 | 10 | 9 | 9 | 10 | 8 |  |
| Malignancy | 23 | 23 | 22 | 22 | 22 | 23 | 17 |  |
| Osteoporosis | 21 | 21 | 21 | 23 | 23 | 18 | 16 |  |
| PTH>6.8pmol/L | 6 | 18 | 9 | 2 | 2 | 4 | 7 |  |
| 25(OH)vitamin D≤25nmol/L | 2 | 9 | 5 | 6 | 6 | 8 | 9 |  |
| 25(OH)vitamin D≤50nmol/L | 19 | 3 | 17 | 14 | 14 | 21 | 15 |  |

^*^In descending order of importance.
